# Dependability of results in conference abstracts of randomized controlled trials in ophthalmology and author financial conflicts of interest as a factor associated with full publication

**DOI:** 10.1186/s13063-016-1343-z

**Published:** 2016-04-26

**Authors:** Ian J. Saldanha, Roberta W. Scherer, Isabel Rodriguez-Barraquer, Henry D. Jampel, Kay Dickersin

**Affiliations:** Department of Epidemiology, Johns Hopkins Bloomberg School of Public Health, 615 North Wolfe Street, Baltimore, MD 21205 USA; Department of Ophthalmology, Johns Hopkins University School of Medicine, 600 North Wolfe Street, Baltimore, MD 21205 USA

## Abstract

**Background:**

Discrepancies between information in conference abstracts and full publications describing the same randomized controlled trial have been reported. The association between author conflicts of interest and the publication of randomized controlled trials is unclear.

The objective of this study was to use randomized controlled trials in ophthalmology to evaluate (1) the agreement in the reported main outcome results by comparing abstracts and corresponding publications and (2) the association between the author conflicts of interest and publication of the results presented in the abstracts.

**Methods:**

We considered abstracts describing results of randomized controlled trials presented at the 2001–2004 Association for Research in Vision and Ophthalmology conferences as eligible for our study. Through electronic searching and by emailing abstract authors, we identified the earliest publication (journal article) containing results of each abstract’s main outcome through November 2013. We categorized the discordance between the main outcome results in the abstract and its paired publication as qualitative (a difference in the direction of the estimated effect) or as quantitative. We used the Association for Research in Vision and Ophthalmology categories for conflicts of interest: financial interest, employee of business with interest, consultant to business with interest, inventor/developer with patent, and receiving ≥ 1 gift from industry in the past year. We calculated the relative risks (RRs) of publication associated with the categories of conflicts of interest for abstracts with results that were statistically significant, not statistically significant, or not reported.

**Results:**

We included 513 abstracts, 230 (44.8 %) of which reached publication. Among the 86 pairs with the same main outcome domain at the same time point, 47 pairs (54.7 %) had discordant results: qualitative discordance in 7 pairs and quantitative discordance in 40 pairs. Quantitative discordance was indicated as < 10, 10–20, > 20 %, and unclear in 14, 5, 14, and 7 pairs, respectively. First authors reporting of one or more conflicts of interest was associated with a greater likelihood of publication (RR = 1.31; 95 % CI = 1.04 to 1.64) and a shorter time-to-publication (log-rank *p* = 0.026). First author conflicts of interests that were associated with publication were financial support (RR = 1.50; 95 % CI = 1.19 to 1.90) and one or more gifts (RR = 1.42; 95 % CI = 1.05 to 1.92). The association between conflicts of interest and publication remained, irrespective of the statistical significance of the results.

**Conclusions:**

More than half the abstract/publication pairs exhibited some amount of discordance in the main outcome results, calling into question the dependability of conference abstracts. Regardless of the main outcome results, the conflicts of interests of the abstract’s first author were associated with publication.

**Electronic supplementary material:**

The online version of this article (doi:10.1186/s13063-016-1343-z) contains supplementary material, which is available to authorized users.

## Background

The results of randomized controlled trials (RCTs) presented at conferences and reported in conference abstracts (“abstracts”) influence clinicians, patients, and systematic reviewers and can affect clinical decisions when abstracts are the sole sources of reported results [1–3]. In addition, when abstracts are not published in full, systematic reviewers are recommended to include the results reported in abstracts [4–7].

RCT information reported in conference abstracts, however, may not be reliable. Abstracts do not typically undergo full peer review, may contain preliminary results, and may contain insufficient information to assess methodological quality [8]. Indeed, differences between abstracts and other sources have been found for results of RCTs [9, 10]. In ophthalmology, 34 % of primary outcomes in abstracts had not been specified as such in ClinicalTrials.gov, a major clinical trials registry [11]. In other fields, results have been shown to be discrepant between abstracts and full publications (“publications”) from the same RCT. Studies in orthopedics [12, 13], cardiology [14], pediatrics [15], pediatric surgery [1], and infectious disease [16] have shown that 40 to 60 % of reports describing RCTs present discrepant results in abstracts and publications. In ophthalmology, however, the extent of discrepancies in the results reported in abstracts and publications is unknown. Additionally, interim or final study results may be reported in abstracts [17], and sometimes, whether the RCT being reported has been completed is unclear. In addition, publishing often is not a goal of abstract authors [18].

Across a number of fields, published RCTs may be fundamentally different from RCTs reported only in abstracts. For example, abstracts describing statistically significant effect estimates or those with positive results have been shown to be more likely to be published in full [19–21]. Industry funding also has been shown to be positively associated with publication [20], and among published RCTs, industry funding is associated with study results that favor the active treatment being evaluated [22–25]. The impact of study investigator conflicts of interest (COIs) on publication is less clear, however. COIs are conditions in which professional judgment concerning a primary interest (e.g., patient welfare or research validity) might be unduly influenced by a secondary interest (e.g., financial gain) [26, 27].

### Objectives

We studied RCTs in ophthalmology to evaluate (1) the agreement in the reported main outcome results between abstracts and corresponding publications and (2) the association between author COIs and the publication of the results presented in the abstracts.

## Methods

### Abstracts

Abstracts were eligible if they described RCT results and were presented at the 2001–2004 conferences of the Association for Research in Vision and Ophthalmology (ARVO). For 2001, we used the printed ARVO abstract book, and for 2002–2004, we used compact discs (CDs) of the ARVO abstracts. We included RCTs addressing any intervention for patients with any clinical condition or involving healthy volunteers.

### Publications

Using two strategies, we searched for the earliest publication (journal article) containing results of each abstract’s main outcome:

1. We searched Medline, Cochrane Central Register of Controlled Trials (CENTRAL), Excerpta Medica Database (EMBASE), Latin American and Caribbean Health Sciences Literature (LILACS), Web of Science, and Scopus. We searched by all abstract authors and unique search terms from the abstract. We searched from 2 years before the abstract was presented through 2013.
2. When no publication was identified, we emailed the abstract’s authors to inquire if the RCT had been published.

### Data extraction

For each abstract and publication, two investigators independently extracted information on author characteristics, study design, participants, interventions, comparisons, and all outcomes. We resolved discrepancies through discussion.

### Classifying statistical significance

For the main outcome (see Table 1 for how this was defined) in each abstract and publication, we extracted all reported data at the last available time point. We considered results for the main outcome as “statistically significant” if the effect estimate, 95 % confidence interval, or *p* value indicated statistical significance at the 5 % level for at least one reported between-arm comparison for the main outcome and “not statistically significant” if no statistical significance at the 5 % level was obtained for any reported between-arm comparison for the main outcome. When insufficient data were reported, or when the authors stated that the results were statistically significant without reporting the effect estimate, 95 % confidence interval, or *p* value, we classified the statistical significance of the results as “not reported.” While our primary analysis focused on the “main” outcome, we also evaluated the statistical significance for “any” (at least one) outcome.

**Table 1:** How we identified the main outcome in each abstract and publication

1. If only one primary outcome was specified, we selected that outcome as the main outcome.	
2. If more than one primary outcome was specified, we selected the first outcome reported in the “Results” section as the main outcome.	
3. If no primary outcome was specified, we selected the outcome mentioned in the “Title” or “Objective” as the main outcome.	
4. If no primary outcome was specified and no outcome was mentioned in the “Title” or “Objective,” we selected the first outcome reported in the “Results” section as the main outcome.	

Because many abstracts did not report statistical significance of the main outcome, we evaluated the association between statistical significance and publication under five different hypothetical assumptions. We assumed the results were statistically significant in none (assumption 1) and in 25 % (assumption 2), 50 % (assumption 3), 75 % (assumption 4), and 100 % (assumption 5) of those abstracts. For assumptions 2, 3, and 4, we used the “runiform” command in STATA^©^ to select abstracts as statistically significant.

### Agreement in the main outcome results between abstracts and corresponding publications

For each abstract/publication pair, we determined whether the main outcome domain was the same and reported at the same time point in both documents. For each pair in which the main outcome domain and time point were the same and statistical significance was reported, we evaluated whether the results agreed.

We classified discordance as either qualitative or quantitative. We defined “qualitative discordance” as a difference in the direction of the effect estimate or the statistical significance of the *p* value. For example, if the effect estimate/*p* value was statistically significant (*p* < 0.05) in the abstract and not statistically significant in the publication (or vice versa), or if one intervention arm was statistically significantly favored in the abstract and another arm was statistically significantly favored in the publication, this would indicate qualitative discordance. We defined “quantitative discordance” of an effect estimate for the main outcome as any difference in the magnitude but not in the direction, when comparing the abstract with the publication. Specifically, for each abstract/publication pair, we calculated the percent difference in the reported effect estimates as follows:

$$ Percent\kern0.24em  Difference=\frac{Effect\kern0.24em  estimate\kern0.24em  reported\kern0.24em  in\kern0.24em  abstract- Effect\kern0.24em  estimate\kern0.24em  reported\kern0.24em  in\kern0.24em  publication}{Effect\kern0.24em  estimate\kern0.24em  reported\kern0.24em  in\kern0.24em  publication}\times 100 $$

This equation for percent difference was applied to all effect estimates (e.g., the relative risk, odds ratio, and hazard ratio). We categorized the quantitative discordance based on the percent differences: < 10, 10 to 20, and > 20 %. When effect estimates were reported in both abstract and publication, we used the reported effect estimates to make the comparison. When effect estimates were not reported, but sufficient information was reported to calculate them, we did so. When only *p* values were reported (without effect estimates or sufficient information to calculate effect estimates), any quantitative difference in the *p* value, without a difference in the direction of statistical significance (at the 5 % level), also qualified as quantitative discordance; we categorized these as “quantitative discordance – amount unclear.”

### Author COIs

We adopted the following COI classification system used by ARVO in 2001–2004: (1) financial support, (2) personal financial interest, (3) employment by a business with interest, (4) consultancy to a business with interest, (5) inventor/developer with patent, and (6) receiving at least one gift from a business with interest in the past year (Additional file 1: Table S1). For 2001, the COI data were presented only as aggregated information for the entire author team. From 2002, ARVO required disclosure of COI separately for each author, and we extracted the disclosed COI for each author.

### Statistical analysis

To evaluate the agreement in main outcome results in the abstract/publication pairs, we calculated the frequency with which each form of discordance occurred.

In model 1, we estimated the relative risk (RR) of publication using log-binomial models to examine the association between the COI and publication. In model 2, we examined whether there was an interaction between statistically significant results for the main outcome as reported in the abstract and the association between the COI and publication. Model 2 contained terms for the interaction between the COI and (1) whether the statistical significance for the main outcome was reported in the abstract and (2) whether the main outcome in the abstract was statistically significant. We ran models 1 and 2 separately for at least one COI and for each specific COI for the first author, last author, and any author.

We plotted Kaplan-Meier curves depicting the cumulative probability of publication of abstracts over time (in months). We conducted log-rank significance tests of differences in the probability of publication at any time point during follow-up [28]. We considered abstracts published before or at the time of presentation at the conference as being published within 1 month of presentation.

## Results

### Specification of the main outcome in the abstracts

For the years 2001–2004, 513/20,721 (2.6 %) abstracts presented at ARVO described RCT results (Additional file 2: Figure S1). We classified the only stated primary outcome as the main outcome for 49 abstracts (9.6 %), the outcome mentioned in the “Title” or “Objective” for 318 abstracts (62.0 %), and the outcome reported first in the “Results” section for the remaining 146 abstracts (28.5 %).

### Agreement in the main outcome results between the abstracts and corresponding publications

**Fig. 1:**
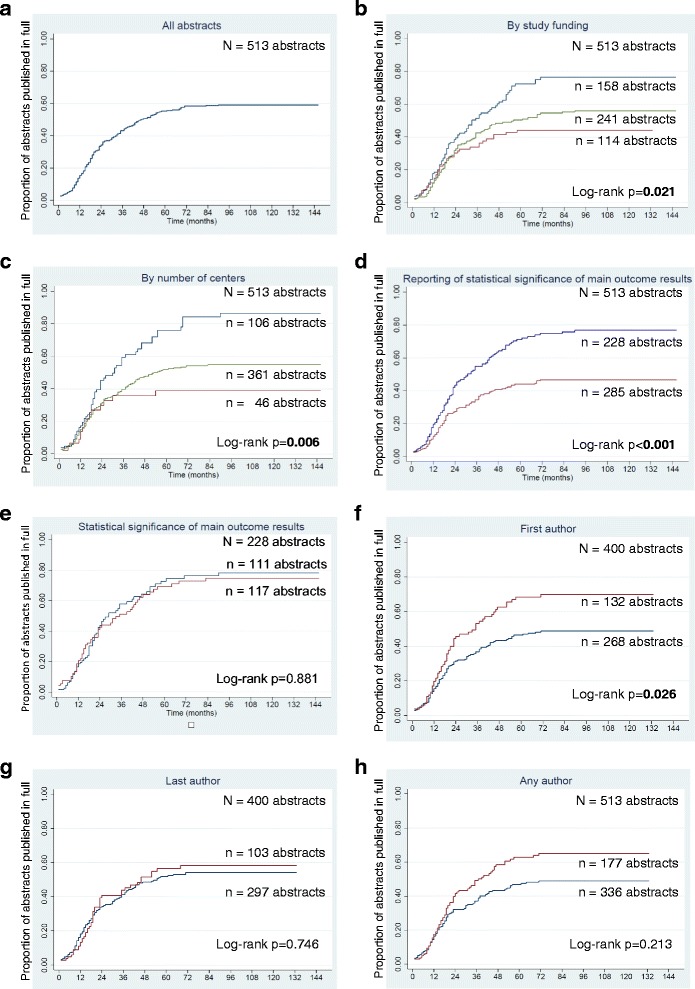
Kaplan-Meier plots showing time to full publication of abstracts of randomized controlled trials (*RCTs*) presented at the ARVO conference during the years 2001–2004, overall and by various study characteristics, author characteristics, and statistical significance of results for the main outcome. **a** All abstracts. **b** By study funding. *Blue* = funded; *green* = not reported; *maroon* = not funded. **c** By number of centers. *Blue* = multicenter; *green* = not reported; *maroon* = single center. **d** By reporting of statistical significance of results for the main outcome. *Blue* = reported; *maroon* = not reported. **e** By statistical significance of results for the main outcome. *Maroon* = statistically significant; *blue* = not statistically significant. **f** By whether or not the “first author” reported at least one conflict of interest (*COI*). *Maroon* = at least one COI; *blue* = no COI. **g** By whether or not the “last author” reported at least one COI. *Maroon* = at least one COI; *blue* = no COI or not applicable/abstract had only one author. **h** By whether or not ANY AUTHOR reported at least one COI. *Maroon* = at least one COI; *blue* = no COI

Of the 513 abstracts, 230 (44.8 %) were published (Table 2). The median time from the conference presentation to publication was 18 months (interquartile range (IQR) = 11 to 33, range = 1 to 90) (Fig. 1a). The main outcome domain was the same for 190/230 (83.6 %) abstract/publication pairs (Additional file 2: Figure S1). For 19 of the 190 pairs (10.0 %), we ascertained that the RCT was ongoing (still following participants) when the abstract was written.

**Table 2:** Characteristics, main outcome results (overall and by whether or not the randomized controlled trial (RCT) described in the abstract was published), and the association with publication of the abstracts of RCTs presented at the Association for Research in Vision and Ophthalmology (ARVO) conferences during the years 2001–2004

Characteristics		All abstracts (*N* = 513)		Abstracts of unpublished RCTs (*N* = 283)		Abstracts of published RCTs (*N* = 230)		Relative risks (RR) (95 % CI)	
		*n* (%**)		*n* (%**)		*n* (%**)			
Characteristics of the RCTs									
Funding									
	Not reported	241	(47.0)	137	(48.4)	104	(45.2)		
	Reported	272	(53.0)	146	(51.6)	126	(54.8)		
	At least one funding source	158	(58.1)	73	(50.0)	85	(67.5)	**1.32**	**(1.09–1.60)**
	Industry (pharmaceutical or other)*	56	(20.7)	22	(15.1)	34	(27.0)	**1.42**	**(1.12–1.79)**
	Government*	71	(26.1)	31	(21.2)	40	(31.8)	**1.31**	**(1.04–1.65)**
	Other*	59	(21.7)	30	(20.6)	29	(23.0)	1.11	(0.84–1.47)
	No funding	114	(41.9)	73	(50.0)	41	(32.5)	**0.76**	**(0.58–0.99)**
Number of centers									
	Not reported	361	(70.4)	208	(73.5)	153	(66.5)	Ref	
	Reported	152	(29.6)	75	(26.5)	77	(33.5)	1.20	(0.98–1.46)
	Single center	46	(30.3)	31	(41.3)	15	(19.5)	Ref	
	Multicenter	106	(69.7)	44	(58.7)	62	(80.5)	**1.79**	**(1.15–2.80)**
Presentation at ARVO									
Poster	418	(81.5)	239	(84.5)	179	(77.8)	Ref		
Oral	95	(18.5)	44	(15.5)	51	(22.2)	1.25	(1.01–1.56)	
Main outcome results									
Main outcome - Statistical significance									
	Not reported	285	(55.6)	178	(63.9)	107	(46.5)	Ref	
	Reported	228	(44.4)	105	(37.1)	123	(53.5)	**1.44**	**(1.19–1.74)**
	Not statistically significant	111	(48.7)	52	(49.5)	59	(48.0)	Ref	
	Statistically significant	117	(51.3)	53	(50.5)	64	(52.0)	0.97	(0.76–1.24)

* More than one option could apply to each abstract

** Percentages are column percentages. Percentages in the shaded rows are calculated using as the denominator number of abstracts reporting that characteristic

Data (RRs and 95 % CIs) reported in bold text are statistically significant at the 5 % level

**Fig. 2:**
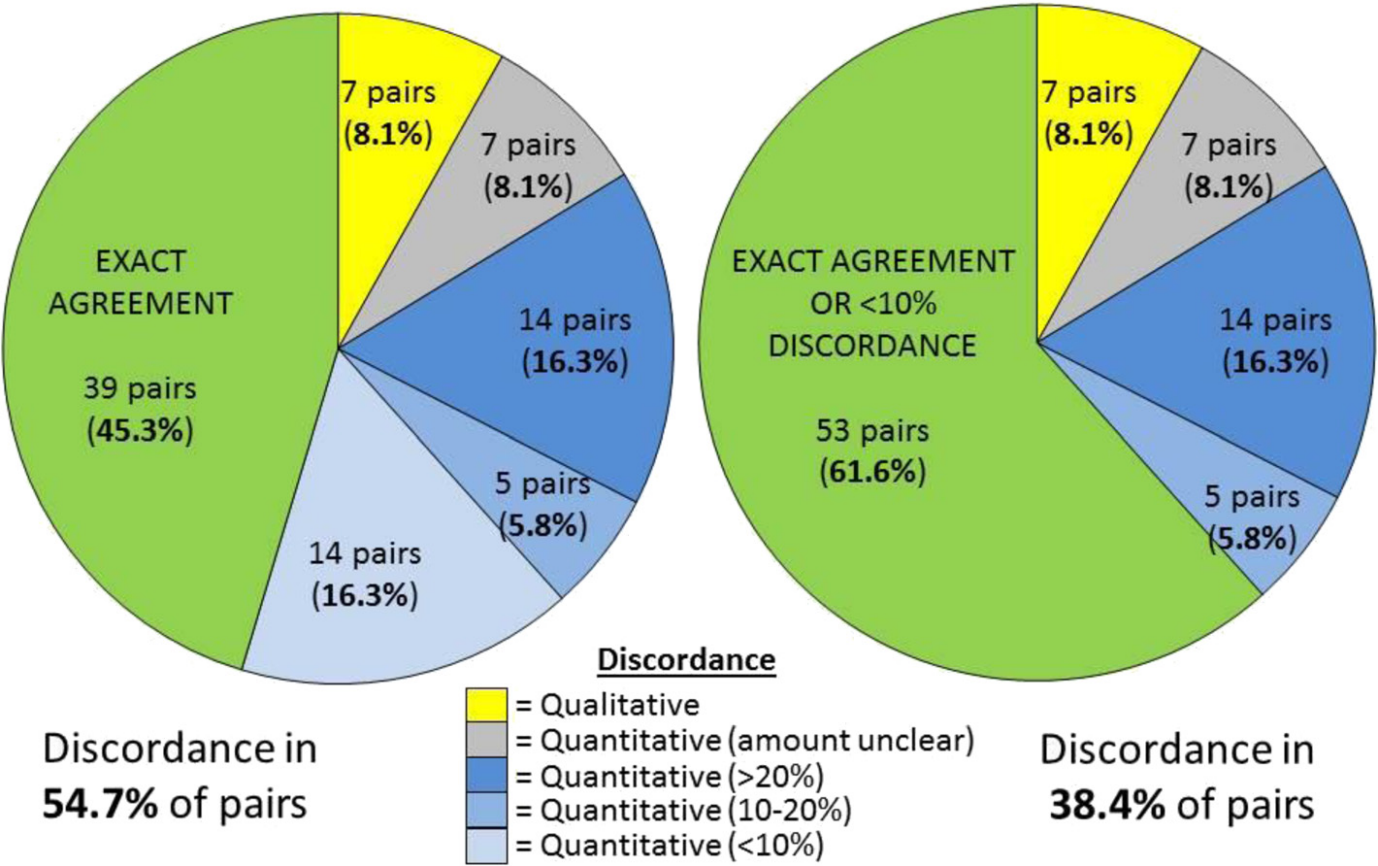
The amount of agreement in the main outcome results in 86 pairs of conference abstracts and full publications. Exact agreement (*green*), qualitative discordance (*yellow*), and various categories of quantitative discordance (*blue*) are depicted under two different definitions of agreement – exact agreement (**a**, *left*) and exact agreement or < 10 % discordance (**b**, *right*)

The main outcome agreed, and the results were reported in both the abstract and the publication at the same time point for 86/190 pairs. The results exactly agreed for 39/86 pairs (45.3 %), and discordance was noted for 47/86 pairs (54.7 %): quantitative discordance in 40 pairs and qualitative discordance in 7 pairs (Fig. 2a). If we defined agreement as exact agreement or < 10 % of discordance, discordance was observed for 33 pairs (38.4 %) (Fig. 2b).

### RCT funding characteristics and publication

The proportions of abstracts reporting industry, government, and other funding were similar (20.7, 26.1, and 21.7 %, respectively) (Table 2). The abstracts reporting no funding were less likely to be published compared with all other abstracts (RR = 0.76; 95 % CI = 0.58 to 0.99), whereas abstracts reporting industry and government funding were more likely to be published. These findings are reflected in the shorter time-to-publication for the funded RCTs compared with all other RCTs (log-rank *p* = 0.021) (Fig. 1b).

### Statistical significance of results and publication

The statistical significance of the results for the *main* outcome was reported in 228/513 (44.4 %) abstracts (Table 2). The results were statistically significant in 117 of the 228 abstracts (51.3 %), and most findings (102/117, 87.2 %) favored the experimental arm. Abstracts reporting statistical significance for the *main* outcome were more likely to be published (123/228) than abstracts not reporting it (107/285) (RR = 1.44; 95 % CI = 1.19 to 1.74), and were published sooner (log-rank *p* < 0.001) (Fig. 1d). Among the 228 abstracts reporting statistical significance for the *main* outcome, no association was observed between the results being statistically significant and publication (RR = 0.97; 95 % CI = 0.76 to 1.24) or time-to-publication (Fig. 1e).

Abstracts reporting statistical significance for *at least one* outcome were more likely to be published (135/249) than abstracts not reporting it for any outcome (95/264) (RR = 1.51; 95 % CI = 1.24 to 1.84). Among the 249 abstracts reporting statistical significance for *at least one* outcome, no association was observed between the results being statistically significant and publication (RR = 1.06; 95 % CI = 0.84–1.35).

### Assumption-based analyses of statistical significance of results and publication

Many abstracts (285/513, 55.6 %) did not report statistical significance of results for the main outcome. When we assumed that the results were not statistically significant in the abstracts that did not mention statistical significance (assumption 1) or in a randomly selected 25 % of the abstracts (assumption 2), the abstracts with significant results were more likely to be published (RR = 1.31, 95 % CI = 1.07 to 1.60 and RR = 1.28, 95 % CI = 1.06–1.55, respectively) (Additional file 3: Figure S2). An inverse association was observed when we assumed the results were statistically significant in 100 % of those abstracts (RR = 0.80; 95 % CI = 0.65 to 0.99) (assumption 5).

### Abstract author COIs

Among the 230 pairs of abstracts and publications, at least one difference in authorship between the abstract and publication was observed in 202 pairs (87.8 %) (Additional file 4: Table S2). At least one author reported one or more COI 34.5 % of the time (177/513 abstracts) (Table 3). Compared with last authors, first authors more often reported financial support (18.8 vs. 9.0 %) and less often reported employment by a business with interest (4.3 vs. 13.9 %).

**Table 3:** Author characteristics of abstracts of randomized controlled trials (RCTs) presented at the Association for Research in Vision and Ophthalmology (ARVO) conferences during the years 2001–2004, overall and by whether or not the RCT described in the abstract was published

Characteristics		All abstracts *n* (%**)		Abstracts of unpublished RCTs *n* (%**)		Abstracts of published RCTs *n* (%**)	
Characteristics of first authors							
Primary affiliation		*N* = 513		*N* = 283		*N* = 230	
	Not reported	8	(1.6)	7	(2.5)	1	(0.4)
	Reported	505	(98.4)	276	(97.5)	229	(99.6)
	Academic	311	(61.6)	156	(56.5)	155	(67.7)
	Industry	31	(6.1)	18	(6.5)	13	(5.7)
	Hospital/Clinic	123	(24.4)	76	(27.5)	47	(20.5)
	Other	40	(7.9)	26	(9.4)	14	(6.1)
Conflicts of interest (COI) (years 2002–2004 only)		*N* = 400		*N* = 229		*N* = 171	
	Not reported	0	(0.0)	0	(0.0)	0	(0.0)
	Reported	400	(100.0)	229	(100.0)	171	(100.0)
	At least one COI	132	(33.0)	65	(28.4)	67	(39.2)
	Financial support*	75	(18.8)	31	(13.5)	44	(25.7)
	Personal finance interest*	4	(1.0)	2	(0.9)	2	(1.2)
	Employee of business with interest*	17	(4.3)	13	(5.7)	4	(2.3)
	Consultant of business with interest*	43	(10.8)	23	(10.0)	20	(11.7)
	Inventor/developer with patent*	8	(2.0)	5	(2.2)	3	(1.8)
	Received gifts within the past year*	36	(9.0)	15	(6.6)	21	(12.3)
	No COI	268	(67.0)	164	(71.6)	104	(60.8)
Characteristics of last authors							
Primary affiliation		*N* = 513		*N* = 283		*N* = 230	
	Not applicable (abstract had one author)	28	(5.5)	8	(2.8)	20	(8.7)
	Not reported	8	(1.6)	7	(2.5)	1	(0.4)
	Reported	477	(93.0)	268	(94.7)	209	(90.9)
	Academic	258	(54.1)	140	(52.2)	118	(56.5)
	Industry	85	(17.8)	39	(13.8)	46	(22.0)
	Hospital/Clinic	106	(22.2)	69	(25.8)	37	(17.7)
	Other	28	(5.9)	20	(7.1)	8	(3.8)
COI (years 2002-2004 only)		*N* = 400		*N* = 229		*N* = 171	
	Not applicable (abstract had one author)	21	(5.3)	8	(3.5)	13	(7.6)
	Not reported	12	(3.0)	6	(2.6)	6	(3.5)
	Reported	367	(91.7)	215	(93.9)	152	(88.9)
	At least one COI	103	(28.1)	56	(26.1)	39	(25.7)
	Financial support*	33	(9.0)	21	(9.8)	12	(7.9)
	Personal finance interest*	1	(0.3)	1	(0.5)	0	(0.0)
	Employee of business with interest*	51	(13.9)	28	(13.0)	23	(15.1)
	Consultant of business with interest*	18	(4.9)	8	(3.7)	10	(5.9)
	Inventor/developer with patent*	5	(1.4)	3	(1.4)	2	(1.2)
	Received gifts within the past year*	14	(3.8)	7	(3.3)	7	(4.6)
	No COI	264	(71.9)	159	(74.0)	113	(74.3)
COIs of any author							
COI		*N* = 513		*N* = 283		*N* = 230	
	Not reported	0	(0.0)	0	(0.0)	0	(0.0)
	Reported	513	(100.0)	283	(100.0)	230	(100.0)
	At least one COI	177	(34.5)	91	(32.2)	86	(37.4)
	Financial support*	99	(19.3)	49	(17.3)	50	(21.7)
	Personal finance interest*	6	(1.2)	4	(1.4)	2	(0.9)
	Employee of business with interest*	76	(14.8)	40	(14.1)	36	(15.7)
	Consultant of business with interest*	66	(12.9)	32	(11.3)	34	(14.8)
	Inventor/developer with patent*	13	(2.5)	8	(2.8)	5	(2.2)
	Received gifts within the past year*	55	(10.7)	23	(8.1)	32	(13.9)
	No COI	336	(65.5)	192	(68.9)	144	(62.6)

* More than one option could apply to each abstract

** Percentages are column percentages. Percentages in shaded rows are calculated with *n* reported as the denominator

### COI and publication – overall and interaction analyses (models 1 and 2)

Abstracts with the first author reporting at least one COI had a greater likelihood of publication (RR = 1.31; 95 % CI = 1.04 to 1.64) (Table 4) and shorter time-to-publication (log-rank *p* = 0.026) (Fig. 1f) compared with all other abstracts. A positive association between COIs and publication was not observed for the last author or for any author, however (Table 4 and Fig. 1g and h). Abstracts with the first author reporting financial support from industry (RR = 1.50; 95 % CI = 1.19 to 1.90) or at least one gift from industry within the past year (RR = 1.42; 95 % CI = 1.05 to 1.92) were more likely to be published compared with other abstracts. Results from models 1 and 2 were similar, and none of the interaction terms was statistically significant (Table 4).

**Table 4:** Associations between conflicts of interests (COIs) of authors of abstracts of randomized controlled trials (RCTs) presented at ARVO conferences during the years 2001–2004 and likelihood of publication of the RCTs, overall model (model 1), and interaction model, stratified by statistical significance of results for the main outcome (model 2)

Author COIs	Model 1**	Model 2***			
	Relative risks (RR) (95 % CI)	RR (95 % CI) among abstracts with main outcome not statistically significant	RR (95 % CI) among abstracts with main outcome statistically significant	RR (95 % CI) among abstracts with statistical significance of main outcome not reported	*P* value of F test of interaction ****
COIs of first author (years 2002–2004 only)	*N* = 400	*N* = 87	*N* = 79	*N* = 234	
At least one COI	**1.31 (1.04–1.64)**	1.13 (0.75–1.72)	1.22 (0.82–1.81)	0.93 (0.63–1.37)	0.73
Financial support*	**1.50 (1.19–1.90)**	1.44 (0.95–2.17)	1.37 (0.92–2.06)	1.11 (0.74–1.67)	0.75
Personal finance interest*	1.17 (0.44–3.14)	–	–	–	-
Employee of business with interest*	0.54 (0.23–1.28)	0.64 (0.20–2.03)	0.96 (0.04–3.92)	0.21 (0.03–1.38)	0.64
Consultant of business with interest*	1.10 (0.78–1.55)	0.87 (0.41–1.86)	1.08 (0.58–2.02)	0.86 (0.52–1.40)	0.69
Inventor/developer with patent*	0.88 (0.36–2.16)	–	–	–	-
Received gifts within the past year*	**1.42 (1.05–1.92)**	1.47 (0.87–2.46)	1.49 (0.97–2.28)	0.91 (0.52–1.60)	0.92
COIs of last author (years 2002–2004 only)	*N* = 400	*N* = 87	*N* = 79	*N* = 234	
At least one COI	1.06 (0.82–1.37)	0.93 (0.59–1.47)	1.06 (0.67–1.70)	0.75 (0.50–1.14)	0.85
Financial support*	0.84 (0.53–1.34)	0.73 (0.36–1.50)	0.81 (0.34–1.97)	0.58 (0.25–1.36)	0.96
Personal finance interest*	–	–	–	–	-
Employee of business with interest*	1.06 (0.77–1.47)	0.89 (0.45–1.75)	1.08 (0.58–2.02)	0.82 (0.51–1.31)	0.76
Consultant of business with interest*	1.32 (0.86–2.03)	**1.73 (1.13–2.64)**	1.56 (0.85–2.87)	0.52 (0.15–1.76)	0.36
Inventor/developer with patent*	0.94 (0.32–2.75)	–	–	–	-
Received gifts within the past year*	1.18 (0.69–2.01)	1.52 (0.83–2.78)	1.01 (0.25–4.12)	0.76 (0.30–1.91)	0.70
Not reported	1.18 (0.66–2.10)	–	–	–	-
Not applicable (abstract had only one author)	1.00 (0.54–1.86)	**1.64 (1.00–2.68)**	1.28 (0.72–2.29)	1.03 (0.50–2.12)	0.68
COIs of any author (years 2001–2004)	*N* = 513	*N* = 111	*N* = 117	*N* = 285	
At least one COI	1.13 (0.93–1.38)	1.01 (0.71–1.45)	1.09 (0.77–1.56)	0.81 (0.59–1.11)	0.72
Financial support*	1.16 (0.93–1.45)	1.02 (0.68–1.53)	1.18 (0.82–1.71)	0.81 (0.55–1.18)	0.83
Personal finance interest*	0.74 (0.24–2.31)	–	–	–	-
Employee of business with interest*	1.07 (0.82–1.38)	0.99 (0.62–1.58)	1.13 (0.66–1.94)	0.80 (0.55–1.17)	0.87
Consultant of business with interest*	1.18 (0.91–1.52)	0.94 (0.52–1.69)	1.25 (0.86–1.81)	0.83 (0.55–1.25)	0.68
Inventor/developer with patent*	0.86 (0.43–1.71)	–	–	–	-
Received gifts within the past year*	**1.35 (1.05–1.72)**	**1.53 (1.03–2.27)**	1.36 (0.90–2.05)	0.95 (0.64–1.42)	0.85

* More than one option could apply to each abstract

** Model 1 – Overall model

*** Model 2 – Interaction model – Results are stratified by whether the main outcome results were not statistically, statistically significant, or not reported

**** F-test of interaction tests the overall statistical significance of the interaction between COI and all interaction terms in model 2. We added two interaction terms in model 2: (1) whether or not results for the main outcome in the abstract were statistically significant and (2) whether or not statistical significance of results for the main outcome was reported in the abstract

Data (RRs and 95 % CIs) reported in bold text are statistically significant at the 5 % level

## Discussion

In this longitudinal study of 513 abstracts of RCTs presented at ARVO, 230 (44.8 %) of which were published, we demonstrated either qualitative or quantitative discordance in results for the same main outcome reported at the same time point for 55 % of the 86 abstract/publication pairs. Abstracts with first authors reporting at least one COI were 31 % more likely to be published and were published sooner, compared with all other abstracts, irrespective of the statistical significance of the result for the main outcome in the abstract.

### Dependability of abstracts

We are concerned about the discordance between the results presented in the abstracts and publications, a discordance now reported for various fields. Our finding in ophthalmology is consistent with previous research showing discrepancies in results for 40 to 60 % of pairs of abstracts and publications in various fields [1, 12–16]. In our study, when the same main outcome was reported at the same time point, 7/86 pairs (8.1 %) reported qualitatively different results for the same outcome in the two reports. This implies that if a decision maker were using the results of an RCT to make a treatment decision, approximately one in 12 decisions would be different, depending on whether the decision was based on the abstract or on the publication. Moreover, our finding that some form of discordance is more common than agreement was based on the abstracts of published RCTs; abstracts of unpublished RCTs might be even less dependable.

Almost half the pairs reported quantitatively different results for the same main outcome. A small discrepancy in RR estimates reported in an abstract/publication pair might not be considered meaningful for an individual trial but could impact meta-analytic effect estimates. While a comparison of reported findings would not be appropriate when the abstract presented preliminary findings and the publication the final results, in our assessment, this was the case only 18.3 % of the time (19/104 pairs).

The current existence of discordance mandates that, when only an abstract is available, systematic reviewers should be cautious about including abstract results, running appropriate sensitivity analyses with and without the information [7, 8]. Second, when both the abstract and publication are available and the data for an outcome are discordant, the authors should be contacted for clarification.

One potential reason for results of a given outcome to be discrepant when comparing an abstract and a publication is that the way the analysis was conducted may differ between the two. For example, the analysis conducted for data reported in the abstract might have been preliminary, and changes to the statistical model may have occurred when the analysis was conducted for data reported in the publication, or the analysis reported in the publication might have been post-hoc. Although we did not examine for the presence of such differences in the analyses of our sample, authors of ARVO abstracts have been reported to present results based on analyses not originally planned [11].

### Suggestions to mitigate the impact of discordance between results presented in abstracts and publications

We have two specific suggestions for mitigating the impact of discordance between results presented in abstracts and publications. First, the scientific community should be made aware when the results presented in an abstract are preliminary. This could be achieved through the addition of a checkbox or other simple feature in the abstract submission system that authors would use to indicate abstracts with preliminary results. Readers of abstracts can thus be alerted to the possibility that a newer version of the results might be available elsewhere, and at the very least, the reader can be cautioned about the possible nonreliability of the results. Second, when authors submit manuscripts to journals, authors should be required to indicate whether the results have been previously presented at a conference, and if so, authors should be required to upload the abstract(s). The submitted abstract(s) should be part of the materials provided to the peer reviewers and editors.

### Low proportion of abstracts published

Our study documents an even lower proportion of full publication of results of RCTs presented in abstracts (44.8 %) than has been previously shown in a Cochrane review (63.1 %) [20]. This lower proportion is despite our study having a longer duration of follow up than the studies in the Cochrane review (up to 146 months versus up to 108 months, respectively).

We do not know of a reason inherent to ophthalmology as a specific field that might explain the low proportion of published abstracts in our sample. One possibility is that ARVO organizers encourage the presentation of works in progress, and ARVO has a very high proportion of submitted abstracts that are accepted. It is possible that few authors are aiming to publish in full [18]. For example, junior investigators may present abstracts as a means to cover travel expenses to the conference. Preparation of abstracts and presentation at a conference may also be encouraged as educational experiences, especially in academic institutions. However, the failure to publish results of RCTs should not be swept under the rug. Because abstracts are often interpreted as scientific contributions and are an indication of when reporting biases may exist [20], conference organizers could require a notation in the abstract submission system for authors to indicate when “preliminary” versus “final” results are being reported.

Failure to publish amounts to research waste and, arguably, to scientific misconduct because it violates the trust that patients place in scientists when giving informed consent [29–33]. Randomized controlled trials, as human experiments, must be held to the highest possible standards, including the full publication of results. Even when published, the results must be reported accurately. Conference abstracts are a form of scientific reporting and are considered by clinicians in their clinical decision-making and by systematic reviewers and guideline developers in their assessment of the body of evidence.

### Significance of results and publication

Although previous research has shown that positive results are associated with both the publication and selective reporting of outcomes (*publication bias* and *outcome reporting bias*, respectively), we did not demonstrate such an association. Considering possible explanations for our findings, it is unlikely that we missed many publications because we employed two strategies to identify publications and followed the abstracts for 110 to 146 months, comfortably exceeding the time by which most abstracts are generally published [20, 34]. Two other possible reasons that may explain what we found are related to missing information. First, more than half of the abstracts did not report statistical significance of results for the main outcome; these abstracts are likely not a random subset of all the abstracts in our study. Indeed, when we subjected our data to different assumptions for the missing information about statistical significance, our results were similar to those reported by others. For example, when we assumed that 25 % of the abstracts not reporting statistical significance were, in fact, statistically significant (assumption 2), abstracts with statistically significant results for the main outcome were 28 % more likely to be published than abstracts with main outcome results not statistically significant. Given that outcomes with positive results are selectively reported and approximately half of all reported main outcomes in our study were statistically significant, we believe it is likely that fewer than half the main outcomes in abstracts not reporting statistical significance were actually statistically significant (i.e., assumptions 1 and 2 are reasonable). Indeed, research has shown that only 43.4 % of unpublished RCTs have statistically significant results [20]. Second, only 9.6 % of abstracts in our study named a “primary” outcome. We developed an algorithm *a priori* to determine the main outcome for the remaining abstracts. Although this algorithm is likely consistent with how most readers of abstracts determine the most important outcome, our algorithm might have influenced our findings.

A tradeoff for our study’s strength of a long duration of follow-up is that the abstracts we examined were presented at ARVO from 2001 to 2004. This period was prior to initiatives such as compulsory registration of RCTs. Mandatory registration of RCTs at ClinicalTrials.gov or at registries within the WHO International Clinical Trials Registry Platform (ICTRP) (http://www.who.int/ictrp/en/) might have mitigated the impact of publication bias and outcome reporting bias in more recent abstract/publication pairs. In addition, the CONSORT for Abstracts extension of the Consolidated Standards of Reporting Trials (CONSORT) Statement includes clear specification of the primary outcome as an essential checklist item [35]. Initiatives like these might have improved the reporting of outcomes in more recent abstract/publication pairs. However, recent studies examining abstracts of RCTs [11, 36–38] and diagnostic test accuracy studies [39] have indicated that the reporting of results in abstracts is still suboptimal.

### COI and publication

COIs are prevalent in biomedical research. Campbell and colleagues, in a survey of academic investigators, reported that approximately 28 % of investigators received financial support, 43 % received gifts, and 33 % had financial ties with industry [40]. Similarly, Rochon and colleagues surveyed 844 biomedical investigators conducting both industry funded and non-industry funded clinical trials, and asked respondents about adherence to 11 prespecified preferred practices known to promote the objectivity of research [41]. Adherence to these practices was reported to be low overall, especially among investigators conducting industry-funded trials. In a separate survey of guideline-development panel members, Neuman and colleagues reported that 52 % of panel members declared some form of financial association with industry [42].

The potential impact of COIs on the likelihood of publication appears complex. On one hand, financial gain might facilitate selective publication of positive findings [43, 44]. On the other hand, financial gain might facilitate publication, irrespective of the study results. Notably, we evaluated COI separately from the study funding source, which was not reported for almost half of our abstracts. ARVO did not require disclosure of study funding during abstract submission.

First authors less often than last authors reported primary affiliation with industry and employment by a business, and more often reported financial support and gifts from industry. Our finding that first author but not last author COIs are associated with publication is intriguing. First, there could be differential disclosure and under-reporting related to their roles related to a particular abstract [45]. Second, financial support for the first authors of abstracts could allow time to be spent on developing a publication, although we may not know the reason for financial support. For example, was it specifically for writing up the results or a more general support? More details about the reason for financial support leading to the COI could have helped us better elucidate this association. Third, industry could have supported academic first authors because of the advantages of working with those viewed as leaders [46–48]. On the other hand, first authors may be more frequently affiliated with academic institutions than last authors because publishing is a key factor in academic advancement. Finally, we considered whether a greater proportion of first authors than last authors were junior researchers within academic institutions. Submission of abstracts is sometimes a means for junior researchers to attend conferences, and publication in full might be less of a priority, especially if they move on to other positions. Because we were unable to examine this possibility directly (ARVO did not collect author rank/position information), we calculated the frequencies of first and last author changes in the publications (assuming when the abstract’s first author was removed or moved to a different position, the first author had a junior rank). The frequencies were similar, however, making this theory unlikely.

## Conclusions

Systematic reviewers should be cautious about including data from abstracts not associated with full publications. In our study, conference abstracts were not reliable reflections of what is reported in the full publication for the main results. Full publication was not, however, associated with the statistical significance of results for the abstract’s main outcome. COIs were associated with the publication of abstracts only for the abstract’s first author.

## Additional files

Additional file 1: Table S1. Definitions for financial COIs as provided by ARVO. (DOCX 13 kb) Additional file 2: Figure S1. Abstracts presented at Association for Research in Vision and Ophthalmology (ARVO) conferences during years 2001–2004. (DOCX 58 kb) Additional file 3: Figure S2. Unadjusted associations between the statistical significance of the results for the main outcome in abstracts of randomized controlled trials (RCTs) presented at Association for Research in Vision and Ophthalmology (ARVO) conferences during years 2001–2004 and likelihood of publication of those abstracts. Results of the observed results and hypothetical analyses are presented under five different assumptions about the proportion of abstracts with statistical significance of results for the main outcome among abstracts not reporting statistical significance of results for the main outcome. (DOCX 52 kb) Additional file 4: Table S2. Differences in authorship order comparing 230 conference abstracts with their corresponding publications. (DOCX 12 kb)

## Abbreviations

ARVO: Association for Research in Vision and Ophthalmology
CD: compact disc
COI: conflict of interest
CENTRAL: Cochrane Central Register of Controlled Trials
CONSORT: Consolidated Standards of Reporting Trials
EMBASE: Excerpta Medica Database
ICTRP: International Clinical Trials Registry Platform
IQR: interquartile range
LILACS: Latin American and Caribbean Health Sciences Literature
RCT: randomized controlled trial
RR: relative risk
WHO: World Health Organization
