# Supplementary material for: Dependability of results in conference abstracts of randomized controlled trials in ophthalmology and author financial conflicts of interest as a factor associated with full publication
Source: Trials. 2016 Apr 26;17:213. doi: 10.1186/s13063-016-1343-z (PMC4845343; doi:10.1186/s13063-016-1343-z)
Supplement: Additional file 1: Table S1. — Definitions for financial COIs as provided by ARVO. (DOCX 13 kb) [file 13063_2016_1343_MOESM1_ESM.docx]

**Table S1: Definitions for financial COIs as provided by ARVO**

| Type | Description |
| --- | --- |
| Receiving financial support | This category includes funding received through employing institution support or from a not-for-profit or competing company, in the form of research funding/services (e.g. protein sequencing) at no cost, support being the subject matter of the presentation/publication. |
| Personal financial interest | This category includes being an investor in a company or competing company other than through a mutual or retirement fund, which provides a product, service, process, or equipment that is the subject matter of the presentation/publication. |
| Employee of a business with interest | This category includes being an employee of a company or competing company with a business interest that is the subject matter of the presentation/publication. |
| Consultant to a business with interest | This category includes being/having been a consultant for a company or competing company with a business interest that is the subject matter of the presentation/publication. |
| Inventor/developer with patent | This category includes being an inventor/developer designated on a patent, patent application, copyright or trade secret, whether or not the patent, copyright, etc. is presently licensed or otherwise commercialized, which is the subject matter of the presentation/ publication or could be in competition with the technology described. |
| Receiving at least one gift in the past year | This category includes having received at least one gift in kind, honoraria, or travel reimbursement valued at over $1,000 in the last 12 months from a company or competing company which provides a product, service, process, or equipment that is the subject matter of the presentation/publication. |
