# Supplementary material for: Dependability of results in conference abstracts of randomized controlled trials in ophthalmology and author financial conflicts of interest as a factor associated with full publication
Source: Trials. 2016 Apr 26;17:213. doi: 10.1186/s13063-016-1343-z (PMC4845343; doi:10.1186/s13063-016-1343-z)
Supplement: Additional file 2: Figure S1. — Abstracts presented at Association for Research in Vision and Ophthalmology (ARVO) conferences during years 2001–2004. (DOCX 58 kb) [file 13063_2016_1343_MOESM2_ESM.docx]

Abstracts presented at ARVO conferences in

2001 (n=5061), 2002 (n=4802), 2003 (n=5248), and 2004 (n=5610)

TOTAL N=20721

Abstracts of RCTs published in full by November 30, 2013 (n=230)

Abstracts with the same “main” outcome domain as the corresponding full publication, but the full publication did not include results at the last available time-point in the abstract (n=104)

Abstracts with the same “main” outcome domain with results reported at the same time-point as the corresponding full publication (n=86)

Abstracts describing results of RCTs (n=513)

Abstracts with the same “main” outcome domain as the corresponding full publication (n=190)

Abstracts of RCTs not published in full by 2013 (n=283)

Excluded abstracts describing only non-randomized comparisons within RCTs (n=32)

Abstracts describing RCTs (n=545)
