# Supplementary material for: Dependability of results in conference abstracts of randomized controlled trials in ophthalmology and author financial conflicts of interest as a factor associated with full publication
Source: Trials. 2016 Apr 26;17:213. doi: 10.1186/s13063-016-1343-z (PMC4845343; doi:10.1186/s13063-016-1343-z)
Supplement: Additional file 3: Figure S2. — Unadjusted associations between the statistical significance of the results for the main outcome in abstracts of randomized controlled trials (RCTs) presented at Association for Research in Vision and Ophthalmology (ARVO) conferences during years 2001–2004 and likelihood of publication of those abstracts. Results of the observed results and hypothetical analyses are presented under five different assumptions about the proportion of abstracts with statistical significance of results for the main outcome among abstracts not reporting statistical significance of results for the main outcome. (DOCX 52 kb) [file 13063_2016_1343_MOESM3_ESM.docx]

Assumption 4: 75% ‘not reported’ are statistically significant

**RR=0.80 (95% CI=0.65 to 0.99)**

RR=1.01 (95% CI=0.82 to 1.24)

RR=1.22 (95% CI=1.00 to 1.48)

RR=0.97 (95% CI=0.76 to 1.24)

**RR=1.31 (95% CI=1.07 to 1.60)**

**RR=1.28 (95% CI=1.06 to 1.55)**

Assumption 5: 100% ‘not reported’ are statistically significant

Observed results

Assumption 1: 0% ‘not reported’ are statistically significant

Assumption 2: 25% ‘not reported’ are statistically significant

Assumption 3: 50% ‘not reported’ are statistically significant
