# Supplementary material for: Dependability of results in conference abstracts of randomized controlled trials in ophthalmology and author financial conflicts of interest as a factor associated with full publication
Source: Trials. 2016 Apr 26;17:213. doi: 10.1186/s13063-016-1343-z (PMC4845343; doi:10.1186/s13063-016-1343-z)
Supplement: Additional file 4: Table S2. — Differences in authorship order comparing 230 conference abstracts with their corresponding publications. (DOCX 12 kb) [file 13063_2016_1343_MOESM4_ESM.docx]

**Table S2:** **Differences in authorship order comparing 230 conference abstracts with their corresponding publications**.

| Status | | Number of pairs  N (%) | |
| --- | --- | --- | --- |
| No change in authorship | | 28 | (12.2) |
| Middle authors changed, FIRST and LAST authors remained unchanged | | 44 | (19.1) |
| LAST author changed, FIRST author remained unchanged | | 52 | (22.6) |
|  | LAST author moved to be a middle author | 26 |  |
|  | LAST author removed | 16 |  |
|  | Abstract had only one author, additional author(s) added | 9 |  |
|  | Abstract had multiple authors, only FIRST author remained | 1 |  |
| FIRST author changed, LAST author remained unchanged | | 33 | (14.4) |
|  | FIRST author moved to be a middle author | 25 |  |
|  | FIRST author removed | 8 |  |
| FIRST and LAST authors swapped positions | | 6 | (2.6) |
|  | No change in middle authors | 1 |  |
|  | With at least one change in middle authors | 5 |  |
| LAST author changed to FIRST author | | 15 | (6.5) |
|  | FIRST author moved to be a middle author | 8 |  |
|  | FIRST author removed | 7 |  |
| FIRST author changed to LAST author | | 15 | (6.5) |
|  | LAST author moved to a middle author | 7 |  |
|  | LAST author removed | 5 |  |
|  | Abstract had only one author | 3 |  |
| Both FIRST AND LAST author changed | | 37 | (16.1) |
|  | Both authors moved to be middle authors | 14 |  |
|  | Both authors removed | 18 |  |
|  | FIRST author moved to be a middle author, LAST author removed | 2 |  |
|  | LAST author moved to be a middle author, FIRST author removed | 3 |  |
